# An Analysis of the Plant- and Animal-Based Hydrocolloids as Byproducts of the Food Industry

**DOI:** 10.3390/molecules27248686

**Published:** 2022-12-08

**Authors:** Robert Waraczewski, Siemowit Muszyński, Bartosz G. Sołowiej

**Affiliations:** 1 Department of Dairy Technology and Functional Foods, Faculty of Food Sciences and Biotechnology, University of Life Sciences in Lublin, Skromna 8, 20-704 Lublin, Poland; 2 Department of Biophysics, Faculty of Environmental Biology, University of Life Sciences in Lublin, Akademicka 13, 20-950 Lublin, Poland

**Keywords:** food byproducts, food processing, waste material, novel hydrocolloids, waste management

## Abstract

Hydrocolloids are naturally occurring polysaccharides or proteins, which are used to gelatinize, modify texture, and thicken food products, and are also utilized in edible films and drug capsule production. Moreover, several hydrocolloids are known to have a positive impact on human health, including prebiotics rich in bioactive compounds. In this paper, plant-derived hydrocolloids from arrowroot (*Maranta arundinacea*), kuzu (*Pueraria montana var lobata*), Sassafras tree (*Sassafras albidum*) leaves, sugarcane, acorn, and animal-derived gelatin have been reviewed. Hydrocolloid processing, utilization, physicochemical activities, composition, and health benefits have been described. The food industry generates waste such as plant parts, fibers, residue, scales, bones, fins, feathers, or skin, which are often discarded back into the environment, polluting it or into landfills, where they provide no use and generate transport and storage costs. Food industry waste frequently contains useful compounds, which can yield additional income if acquired, thus decreasing the environmental pollution. Despite conventional manufacturing, the aforementioned hydrocolloids can be recycled as byproducts, which not only minimizes waste, lowers transportation and storage expenses, and boosts revenue, but also enables the production of novel, functional, and healthy food additives for the food industry worldwide.

## 1. Introduction

The food industry produces many by-products worldwide. Approximately 38% of waste comes from food processing [1] specifically approximately 20% from meat, fish, and poultry, approximately 4% from dairy (mainly whey—50 million m^3^ yearly), 33% from oil crops, and 35% from fruits, vegetables, and tubers industries [2]. Torres-León et al., [3] claim that waste from fruit processing exceeds 50%—namely bagasse, peels, trimmings, stems, shells, bran, and seeds. A lot of seemingly useless solids and liquids come from plant-based food manufacturers. Food waste can cause environmental issues and generate additional management, storage, and processing costs. Food byproducts are often processed into fodder. Such fodder consists of cereal industry waste, such as rice bran, maize and wheat seeds, husks, hull, banana peels, or feathers. Byproducts not suitable for animal feed, such as onion peels and roots or excess banana peels, are being disposed of [4]. However, many food byproducts can be used instead of discarded. They contain valuable polysaccharides—dietary fiber fractions pectin, chitosan, cellulose, hemicellulose, lignin, and gums; proteins, e.g., single-cell protein of yeast, proteins obtained from de-oiled sunflower press cake, for example, β-lactoglobulin, α-lactalbumin, immunoglobulin, bovine serum albumin, lactoferrin, and lactoperoxidase; lipids with high levels of unsaturated fatty acids; ω-3 PUFAs; natural colorants—apple pigments, anthocyanin-based pigments; bioactive compounds such as citric and linoleic acids, tocopherols, δ-Tocotrienolfunctions, dihydrochalcones, flavanols, polyphenols, ascorbic and phenolic acids, isorhamnetin-O-(di-deoxyhexosyl-hexoside); or hydrocolloids, e.g., starches, glucosides, proteins, gums, and fiber [1,5]. The addition of these compounds may modify the structure of other food products desirably, contribute to functional value, and provide additional income for the industry. Some of the most valuable byproducts, considering texture modification, are starches and gelatin [6].

Starches are renewable polysaccharides that naturally form in most plants, serving a nutritional backup purpose. These biopolymers may be found in plants’ rhizomes, branches, fruits, seeds, and tubers. Starch’s two separate components are amylose and amylopectin [7]. Both compounds contain D-glucose chains; however, they are connected in various ways. Amylose consists of unbranched glucose units linked with α (1–4) glycosidic bonds. Amylopectin also consists of glucose, but it is heavily branched, and the units are linked with α (1–4) glycosidic and α (1–6) glycosidic bonds. Native starches consist of about 10–40% amylose and 70–90% amylopectin. The ratio of those two polysaccharides is unique to individual starches and therefore is responsible for starch’s properties [8]. The weight, shape, and scale of amylose and amylopectin molecules define differences in pasting, retrogradation, rheology, and viscoelastic properties [9]. Exposing starch granules to a specific temperature and moisture makes its structure undergo several changes such as swelling by absorbing water, decreasing the level of crystallinity by amylopectin double helix dissociation, elution of amylose into the aqueous phase, and fracturing starch granules. These changes are known as starch gelatinization [10]. The reverse process, where amylose and amylopectin partially regain their ordered structure, is termed starch retrogradation [11]. Starches derived from tubers and roots require a relatively low temperature to gelatinize, the process is quick, and granules swell uniformly [12]. Moreover, root and tuber starches show a higher viscosity profile and paste clarity than grain starches, yet they tend to retrograde easily. Amylose to amylopectin ratio is the reason for these unique physicochemical properties. Almost all of these starches show B-type X-ray patterns [13].

Gelatin is a protein primarily obtained from the animal industry byproducts—pig skin, bovine and porcine cartilage, bones, and hides during partial hydrolysis of collagen. Collagen is the most common structural protein in animals’ bodies, making up about 30% of all proteins. Animal species majorly influence gelatin properties and tissue types. They are obtained from [14,15]. There are two main uses of gelatin in the food industry: to modify the texture by water binding, providing creaminess and foam, fining [16], gelling, stabilizing, emulsifying, altering the viscosity, or to produce packaging films and coatings, which inhibit the environmental impact on food, prolonging the shelf life. Moreover, the addition of gelatin affects the aroma of food products. An increase in viscosity impedes volatile aroma compound penetration from the inside to the outside of a food product [15]. In addition, the firmer the gelatin gel, the harder it is for aroma substances to be released [17].

This work focuses on the novel utilization of plant and animal food industry byproducts. The byproducts’ physicochemical characteristics, health-promoting benefits, and use as hydrocolloids have been discussed. It is important to establish how many valuable compounds are present in the seemingly useless waste. This way, such ingredients can be identified and extracted or obtained, providing additional income, reducing storage and transportation costs, and mitigating harmful impacts on the environment. The information gathered in this work aims to clarify the topic of novel hydrocolloids from byproducts and show their use in food technology.

## 2. Description of Arrowroot, Kuzu, Sassafras, Sugarcane, Acorns, and Gelatin Byproducts Utilization

### 2.1. Arrowroot

**Figure 1:**
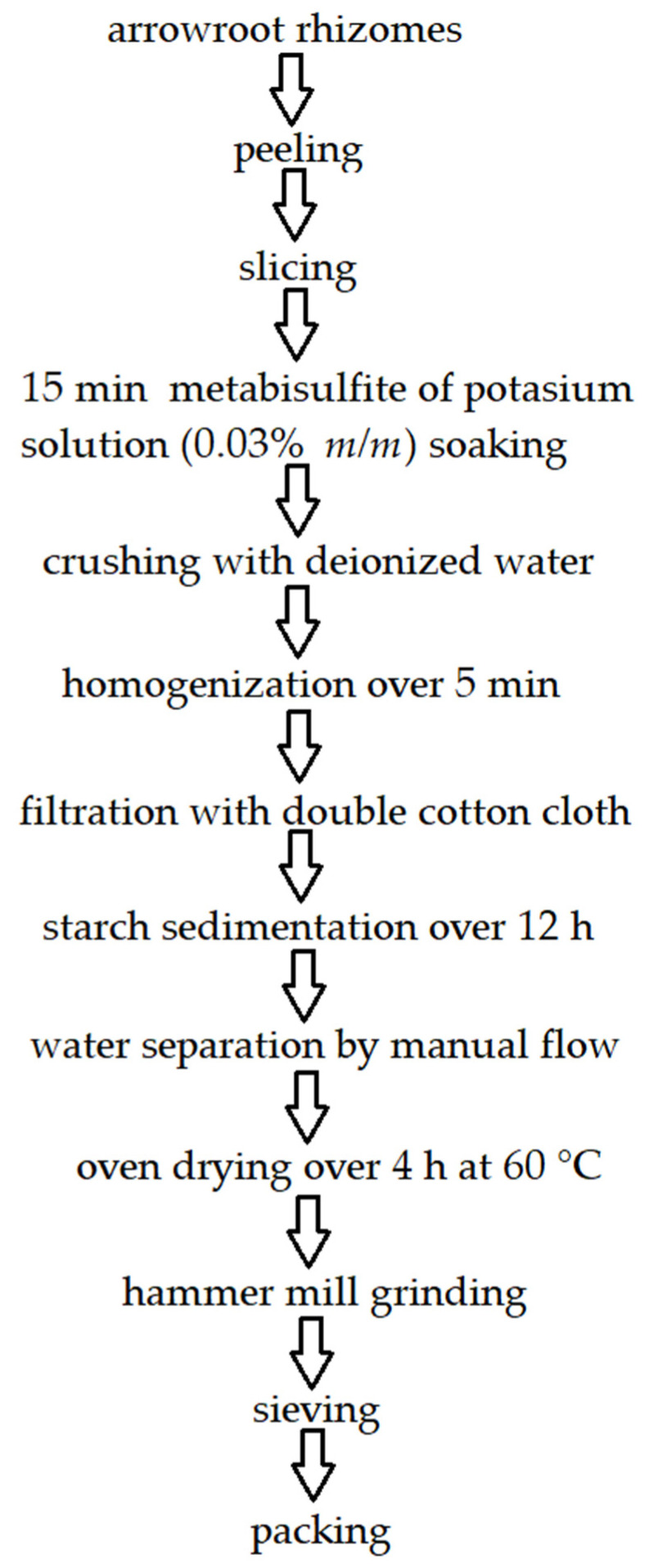
Arrowroot starch extraction. Own figure based on Ref. [29]. 2022, Tarique et al.

Arrowroot *(Maranta arundinacea)*, also known as sago banban, sago rare, sago andrawa, sagu, Patat, arut, jelarut, irut, larut, labia walanta, or hudasula [18], is a native plant to the West Indies [19], Indonesia [20], or tropical regions of South America [21]. The physiochemical properties of arrowroot from various sources are quite similar [22]. Arrowroot can reach 0.9–1.5 m in height [23]. Its flowers are white, and its leaves are big, green, and 10–20 cm long. Rhizomes are fleshy, cylindrical, and tuberous with a width of 2.5–3 cm and 20–40 cm long [24]. The plant’s roots are long and abundant in fibers [25]. Its promising properties were introduced worldwide over colonization times, then the export of starch from tubers and rhizomes outside India began. Arrowroot is commonly cultivated in the Philippines as a perennial crop and used in various bakery products, e.g., Spanish shortbread *polvoron* or pancake topped with grated, young coconut flesh-*saludsod.* The direct consumption of arrowroot rhizome by humans is unclear, because of its very fibrous texture [26]. Arrowroot starch is used instead of many grain flours because of no gluten content [19] and is safe for people with celiac disease, gluten intolerance, and FODMAP (fermentable, oligosaccharides, disaccharides, monosaccharides, and polyols) sensitivity [21]. Three suitable cultivars are cultivated in Brazil: common, creole, and banana [27]. Arrowroot is mainly used for starch extraction, shown in Figure 1, because of its high content in plant rhizomes [28]; however, plant fibers also found utilization as packaging and tissue paper [29]. Arrowroot starch may also produce edible films [30]. Considering various purposes of arrowroot, in some fields such as fiber gathering or pro-health substances extraction, the starch is a byproduct that can be reused.

The plant’s rhizome starch composition is presented in Table 1 and Table 2.

The likely reason for the differences in the starch composition is the plant’s age. Extraction of amylaceous fractions carried out on 12- and 14-month plants indicated an increase in amylose content from 17.9 to 20.0%, respectively. Moreover, starch granule size increased with the plant’s development. On the other hand, the values of viscosity (peak, breakdown, final, and the tendency of retrogradation) decreased as the plant got older [39]. A large amount of amylose, such as 20–30%, is beneficial in gelatinization, lowering the energy required to start the process. Starches with higher amylose content have fewer crystalline regions and lower gelatinization temperatures [21].

The granules’ size is 7–16 μm. Arrowroot starch exhibits a high purity of over 99% [12]. However, Guilherme et al. [26] indicated that arrowroot starch has a high amount of carboxylic acid, suggesting contamination problems and possible unwanted fermentation [40,41]. Microscopic analysis of arrowroot starch showed that granules are circular, ellipsoid, and oval, and their sizes vary [30]. Starch gelatinizes at 63.94 °C and has a B-type crystalline structure [38].

Arrowroot starch exhibits significant thickening, stabilizing [29], and shear thinning properties and may be used as a fat replacer in food [42]. Cassava and potato starches may be fortified with arrowroot starch, to increase the final gel’s stability [12]. Since arrowroot starch is tuber-derived, its granules swell fast and evenly and have a high viscosity profile, surpassing grain starches [12,43]. Numerous physicochemical properties suggest that arrowroot can be mixed with other starches, e.g., to improve resistance to retrogradation and thermal and freeze-thaw stability of the whole composite [12]. Arrowroot starch is found in biscuit, cake, pudding, oatmeal, pie filling [29], soup, candy, condiments, pudding, and ice cream production. Moreover, it can replace wheat flour being a safe alternative for people with celiac disease [18]. Arrowroot starch appears to be a good ingredient for the extrusion process, exhibiting a high expansion ratio and low bulk density in the final product. The extrusion process makes arrowroot starch absorb more water and oil, which is a common phenomenon among starches. Products with extruded arrowroot starch exhibit desirable texture and color. Moreover, the extrusion process lowers the digestibility of the arrowroot starch, by making it more resistant, compared to native starch, which is sought after by people on low-calorie diets [44]. Arrowroot starch is a novel material for the walls of microcapsules. Arrowroot-enriched microcapsules indicated sufficient oxidative stability, shelf life, encapsulation efficiency, low water activity (0.05–0.23), and were hermetic. Moreover, arrowroot starch acted as a cryoprotectant during freeze-drying [45]. Since Arrowroot starch exhibits antioxidative properties [21], it is expected to inhibit oxidation of lipids, thus may be used to prolong the shelf life of products that contain fats such as biscuits, pastries, margarine, a plant-based mayonnaise, and so on [46].

Despite various advantages, native arrowroot starch has some industrial limitations. Due to significant viscosity and discord with some hydrophobic polymers, it has finite solubility and unsatisfactory processability. Due to the immensely low phosphorus content (4.6 nmol mg^−1^), its impact on arrowroot starch is insignificant, limiting some gel and paste behavior modifications [22]. To use the full potential of arrowroot starch, it needs modifications to improve its hydrophobicity, crystallinity, and stability to enzymatic and thermal degradation [47]. Gamma radiation treatment on arrowroot starch resulted in increased breakdown value, pointing to the low stability of the starch granules and low setback value, indicating high resistance to retrogradation thus, suggesting the arrowroot starch utilization for cold and frozen food products [48].

### 2.2. Kuzu

Kuzu, also known as kudzu, kudzuvine, kudsu, wa yaka, aka, nepalem, Japanese arrowroot, kudzu comun (Spanish), vigne japonaise (French) and kopoubohne (German) [49] is a bulbous, climbing shrub in the *Fabaceae* family of *Pueraria* genus native to Asia (China, Japan, Korea, Thailand, Vietnam, and Taiwan) and Malesia (Indonesia, Malaysia, Papua New Guinea, and the Philippines). The most popular kuzu variety is *Pueraria montana var lobata* [50]. Eastern Asians have been using kuzu for several years to create functional properties of food. Starch extracted from kuzu can form a clear, colorless, high-strength gel [8]. Kuzu starch is used as a food stabilizer, microencapsulated wall material, raw material for edible films, texture modifier, and emulsifier [51]. However, emulsifying properties were proven poor compared to protein or surfactants [52]. This plant has also been used as fodder and has various medical purposes [53,54]. Kuzu starch is also used in bioplastic production, constituting nontoxic, biodegradable, transparent, slightly reddish/yellowish material [55]. Kuzu root contains oleanene-type triterpenes and triterpenoid glucosides, including kudzusaponin, kudzusapogenol, and soyasapogenol [56,57,58] fragrant components namely methyl palmitate, methyl stearate, 2-methoxyethyl acetate, acetyl carbinol, and butanoic acid responsible for gently sweet and fruity-wine aroma [59] and a minimal amount of minor constituents such as 5-methylhydrantoin, tuberosin, choline chloride, acetylcholine chloride, D-mannitol, glycerol 1-monotetracosanoate [60], eicosanoic acid, hexadecanoic acid, tetracosanoid acid-2,3-dihydroxypropyl ester [61], diacetonamine, and D-(+)-pinitol [62].

**Figure 2:**
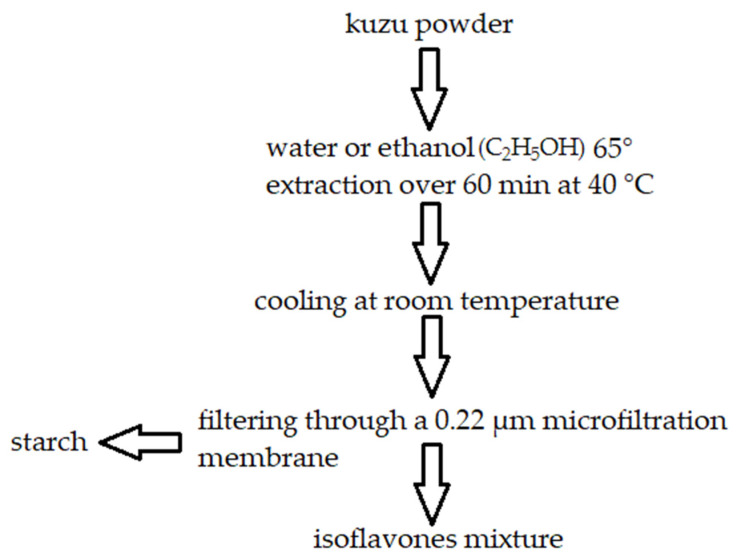
Extraction of kuzu starch and isoflavones. In this method, starch is a by-product. Own figure based on Ref. [64]. 2022, Mocan et al.

A common method of isolating kuzu starch is the standard precipitation method, shown in Figure 2. Since starch and isoflavones have low water solubility, they coexist after precipitation [63] (C_2_H_5_OH).

The kuzu starch composition has been presented in Table 3.

Studies indicate that kuzu has C-type starch [67,68,69]. However, kuzu starch obtained in Vietnam was of A-type and in Korea—B-type [70]. The differences might be due to the genotype and growing conditions [66]. The average degree of polymerization (DPn) of kuzu starch amylose is 1905 and of amylopectin—2017. Amylopectin’s average chain length (CL) is 21, shorter than that of amylose, 151. However, the mean number of chains per molecule (NC) of amylose is 12.6, while that of amylopectin is 96.4 [68]. According to Van Hung and Morita [71], the region where kuzu is cultivated influences DPn, CL, and NC. The largest DPn and NC of amylose and amylopectin is established for kuzu from Vietnam. The longest CL of amylopectin molecules (30) was found in kuzu starch from Japan. The highest CL of amylose (236) exhibited starch from Korea. The composition of isoflavones varies depending on the cultivars, growing regions, isolating techniques, plant’s growth phase [72], where starch was obtained, and if the process was carried out commercially or at home. The highest concentration of daidzein and daidzin was found in Korea—16.41 mg/100 g starch, whereas starch from Japan had 2.18 mg of daidzein per 100 g of starch [71]. Starch is located in the plant’s roots [73]. Starch manufactured commercially has no daidzin, genistein, genistin, or puerarin. Daidzein was present with a concentration of 0.011 mg/g dry basis. In the homemade sample, all the mentioned isoflavones were present with a total concentration of 8.277 mg/g dry basis [74]. Given the medical use of the mentioned bioactive compounds, effective purification and separation from the starch are essential. In the aftermath, starch is considered a byproduct, which should not be discarded due to its significant value and may be used in the food industry.

Kuzu starch exhibits a high lightness value (L*) of 93.34, which makes it a desirable product [67,75]. Granules are irregular, polygonal, spherical, and hemispherical and have a smooth surface without cracks [76] and a diameter of 3 to 23 µm. On average, the amylopectin molecule weight is 2.05 × 108 Da, and of amylose, it is 1.89 × 106 Da [69]. The starch has a polysaccharide structure with a high pasting temperature of 70–76 °C. It is rich in micronutrients such as phosphorus, iron, and calcium, providing a valuable alternative to thickening and binding agents such as gelatin [77]. Kuzu starch is used to manufacture edible films, stabilize emulsions, encapsulate oxidizable functional substances, modify the texture of food, and develop functional foods [78,79,80]. Native kuzu starch is used worldwide as a pro-health ingredient in foods such as nutritional powders, beverages, noodles, or vermicelli [81]. The functional properties include transparency, solubility, swelling power, freeze-thaw stability, gelatinization, retrogradation, pasting property, dynamic rheological property, and in vitro digestion [66]. Kuzu starch exhibits a transparency of 50.6 [82], indicating high phosphorus content [83]. The solubility is 8.55%, and swelling power is 3.95% at 50 °C. The increase in temperature to 90 °C caused an increase in solubility and swelling power—the reason being the loosening of chemical bonds in starch granules. Native kuzu starch has poor syneresis resistance. After five freeze-thaw cycles, the percentage of syneresis increased from 12% to 52.56% [84]. Starch content (*w*/*v*) affects gel structure. 0.1% concentration makes the gel resemble homogeneous, stubby, and curved strands, while 2%—thick masses and entangled aggregates. Storing gels at 4 °C for a week caused the gels to be retrograde, creating an opalescent surface and, at a concentration of 2%, fibrous clusters [85]. To establish kuzu starch thermal properties, differential scanning calorimetry (DSC) is mainly used [66]. Native kuzu starch shows higher gelatinization temperatures and enthalpy than retrograded kuzu starch due to fewer crystalline regions [69]. Inappropriate chain length hinders the retrogradation of starch molecules. The best length for starch to retrograde is 14–24 degrees of polymerization [86]. Most molecules of kuzu starch have a degree of polymerization ranging from 13 to 24, making kuzu starch a starch of a high retrogradation degree (RD)—44.4% [69]. However, kuzu starch RD is higher the lower the temperature is. The reason is starch molecules diffusion and lower nucleation of the molecules. Additionally, incorporating saccharides or sodium chloride may alter kuzu starch RD based on its storage temperature [87]. To prevent starch retrogradation, tea polyphenols and catechins might be applied [88]. Compared to potato starch, canna starch, fern starch, and adzuki bean starch, kuzu starch exhibits the greatest pasting temperature because of its little starch granules, limiting swelling capacity at high temperatures [67,69]. Kuzu starch pasting abilities might be modified by adding other hydrocolloids, e.g., xanthan gum and soluble soybean polysaccharide. The reason is interactions with leached amylose or amylopectin from starch granules [66]. Kuzu starch exhibits shear thinning behavior when the content is not less than its Ce [85]. As kuzu starch concentration increases, so do the storage modulus (G′), loss modulus (G″), and shear viscosity. Additionally, adding sodium chloride, sucrose, and maltodextrin can modify the rheological properties [89]. Regarding the application, kuzu starch should be treated differently. The best conditions to prepare kuzu starch pastes for application in the food industry are temperatures of 80 °C and 15 min time; for the pharmaceutical industry—95 °C and 75 min; for the cosmetic industry—80 °C and 30 min [90].

Raw kuzu starch shows moderate in vitro digestibility. After 120 min of exposure to digestive enzymes, many eyelets were noticed on the surface of kuzu starch granules. Kuzu starch exhibits a rapidly digestible starch (RDS) content of 5.66%, slowly digestible starch (SDS)—of 25.88%, and 68.46% of resistant starch (RS). Such properties suggest that kuzu starch might be used as a functional ingredient for lowering the glycemic index in food. However, after gelatinization, RS lowered to 11.38% because of heat-damaging crystalline regions, making starch chains easier for amylase to interact with [91,92]. The percentages of SDS and RS can be raised by heating them for 1–24 h at the temperature of 50 °C causing amylose-amylose and amylose-amylopectin interactions to amplify, probably making starch chains “temper” and more resistant to enzymes [15,93]. Another method of limiting kuzu starch digestibility is a fortification with xanthan gum (1–2% *w*/*w*). Xanthan gum tends to adsorb on the starch granules, providing a defense against digestive enzymes [94].

Natural extracts present in kuzu flour limit the increase in crystallinity and recrystallization of starch [8]. There is inaccuracy in the case of the kuzu starch level of crystallinity. Starch from Vietnam exhibits a degree of crystallinity of 38.6%, Japan—35.9%, and Korea—35.7% [70]. According to Wang et al. [68], relative crystallinity is at a level of 35.25%. Reddy et al. [67] suggest 23.45% confirmed by X-ray diffraction analysis. The most likely reasons for these contractions are the kinds of cultivars, sample growing conditions, or quantification methods [95].

Most isoflavones are destroyed while starch is extracted from the roots. Thus, another isolation method is advised [66].

Native kuzu starch has some industrial limitations, such as low solubility or low stability; thus, some structural modifications might be needed [66]. Kuzu starch may be modified physically, including annealing or extrusion treatment [96,97]. Annealing involves heating the starch granules to a temperature between the glass transition and the initial gelatinization for a given time in an aqueous environment [98,99]. Heating at 50 °C for 1–9 days does not change the C-type of kuzu starch, but the ratio of B-type polymorphs grows compared to non-modified starch. What is more, annealing caused an increase in gelatinization temperatures, enthalpy, pasting temperature, and prior stability and a reduction in pasting viscosities, granular swelling power, and solubility because of internal rearrangement and interplay of starch particles [97]. Furthermore, annealed kuzu starch might have health benefits for individuals who require lowered digestibility of food—the SDS and RS percentage in tempered kuzu starch is 10% higher compared to native kuzu starch. The reason is amplifying interplay between amylose and amylose or amylopectin after tempering [93]. Annealed kuzu starch might find use in manufacturing canned and frozen foods because of lowered swelling power, solubility and increased paste stability and crystallinity. Moreover, lowered granular swelling and amylose leaching and increased heat and shear stability suggest utilization in noodle production [97]. Enzymatic modification utilizing α-amylase and transglucosidase may be used to address the inferior pasting qualities and propensity for retrogradation of kuzu starch. This way, modified kuzu starch exhibits higher solubility, paste clarity, gelatinization temperature, and lower viscosity due to slower retrogradation [81]. Another method of kuzu starch modification is esterification with octenyl succinic anhydride. Such treatment improves emulsification properties, viscosity, and granule swelling compared to native kuzu starch [52]. According to Chen et al., [98], it is possible to cross-link kuzu starch using sodium trimetaphosphate, which grants the starch more desirable thermal, freeze-thaw, and retrogradation stability, and higher viscosity. This may prove useful in jelly, jam, gummy candy, mayonnaise, preserves, sauces, instant meals, and pastry production. Kuzu starch modified with dodecenyl succinic anhydride exhibits larger granule size, higher viscosity, lower gelatinization temperature and enthalpy value. Starch modified this way shows better emulsification properties compared to native starch and may be used as a wall stabilizer in the production of microcapsules filled with oil or bioactive compounds [99].

### 2.3. Filé Powder

*Sassafras albidum* is a deciduous tree species native to North America. The tree’s root was used in folk medicine and as a spice in soft drinks such as root beer but was prohibited in 1960 because of an unsafe amount of carcinogenic alkaloid safrole (4-allyl-1,2-methylenedioxybenzene). In the present day, safrole-free extracts are allowed to be used as flavorings. Many other alkaloids were found in the roots, but none in the leaves [100].

Filé powder is a spice and a thickening agent made of young, dried, and ground Sassafras tree (*Sassafras albidum*) leaves; however, Parekh [101] refers to filé powder as ground sassafras root. Filé powder is a crucial ingredient of Creole gumbo [102]. Originally gumbo was made using okra instead of filé powder by l Choctaw Native Americans. Filé powder was introduced later and proven useful when okra was out of season [103]. At high temperatures, filé powder thickens unusually, forming unappetizing gelatinous strings [104].

Parts of the sassafras tree have many uses. Orange-wood is used to make barrels, buckets, posts, and furniture; oil is used in perfume and soap production; the drinkable brew is made of roots’ bark [105]. However, sassafras bark hot water infusions are not recommended due to the harmful safrole content and may interfere with medicine intake [106]. Considering the processing of sassafras trees, the leaves are a byproduct, which may be used in the production of food additives instead of being discarded.

Given the thickening properties of filé powder and its herby taste, it is expected to find use in instant soups and meals, salty sauces, meat pies, vegetable pastes, loaves, bread spreads, cocktail mixers etc. To the authors’ best knowledge, there is very little information about filé powder utilization in food technology.

### 2.4. Sugarcane

Sugarcane places among the most valuable crops in food and energy industries. Sugarcane production in 2020 reached 1.9 billion tons [107]. Its notable trait is to accumulate large content of sucrose in its stems and a very high yield of 80 tons/ha but in theory, it is possible to achieve over 380 tons/ha due to the breeding programs or gene engineering. Sugarcane is mainly cultivated for sugar and, following, ethanol production [108]. The sugarcane industry is responsible for a lot of waste. For every 1 ton of sugar produced, 9 tons of byproducts are generated—3–3.4 tons of bagasse, 4.5 tons of molasses, 0.3 tons of filter (press) mud, and, consequently during the manufacturing process, 12 tons of fumes. These byproducts are abundant in carbon compounds and minerals, thus may be used for extraction, physicochemical transformation, or fermentation to fortify products such as construction materials, drugs, substrates for enzymes in the production of chemicals, food and fodder, pesticides or to obtain fiber, low-calorie sweeteners, vitamin acids, beverages, oils, protein, fodder, fertilizer (press mud), and fuel. However, there are byproducts of lesser commercial value which are trash, green tops, wax, fly ash, and spent wash. Numerous organizations in leading sugar-producing nations such as Australia, Brazil, Cuba, Mauritius, Taiwan, South Africa, China, and India have been revolutionized into “Sugar Complexes” which supplied not only sugar but also waste-derived products due to the abundance of economic opportunities in the production of sugarcane byproducts [109,110].

**Figure 3:**
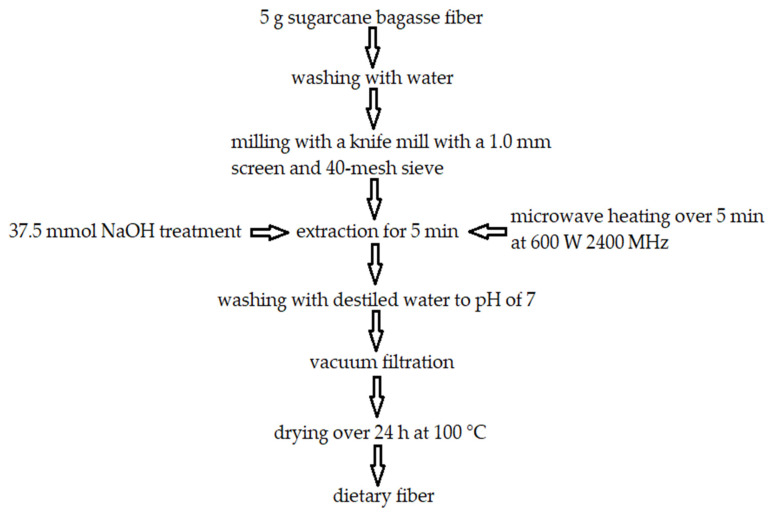
Production of dietary fiber from raw sugarcane bagasse fibers. Own figure based on Ref. [119]. 2022, Gil-López et al.

Bagasse is a fibrous waste generated after sugarcane is crushed during sugar production. Fresh bagasse has a high moisture content of about 50% and is later dried to a composition of 45% cellulose, 28% pentosans, 20% lignin, 5% sugar, 1% minerals, and 2% ash [110], however, Paturau, [111] reports 55–58% cellulose, 26–32% hemicellulose, 19–22% lignin, and Sangark and Noomhorm [112], claim 45% cellulose, 26% hemicellulose, and 19% lignin. Bagasse is a complex carbohydrate biopolymer structure. Monomers of these biopolymers are connected by four bonds, namely ether, ester, hydrogen, and carbon-carbon bonds [113]. Functional groups are connected via ether bonds, hemicelluloses, cellulose, and lignin are connected via ester bonds, hydrogen bonds are present in carbon-carbon structure in the aromatic rings and the cellulose polymer chains, β-1,4 glycosidic bonds are present between long, linear homopolysaccharide of anhydroglucose and the cellulose fractions. Bagasse cellulose molecular mass is 157,800 to 168,400 g/mol and the cellulose fibers are 1–1.5 mm in size. Bagasse is resistant to enzymatic and chemical hydrolysis due to cross-linkage between hemicellulose matrix and micro- and macrofibrils of cellulose and due to the degree of polymerization, which depends on glucose units in a polymer. The crystallinity index of bagasse is established as 56.7% [114]. Bagasse hemicellulose consists of β-1,4 xylopyranose backbone, β glucans, xyloglucans, glucomannans, galactomannans, and scarcely of uronic acids. Bagasse lignin‘s weight averages from 507 to 3973 mol/g. Syringic acid, ferulic acid, vanillic acid, p-coumaric acid, xylose, glucose, arabinose, galactose, acetosyringone and syringaldehyde are present in bagasse lignin fractions [115]. Dried bagasse composition is similar to that of wood. Approximately half the generated dried bagasse is sufficient to provide the sugarcane processing unit with energy and ethanol–fuel. The leftovers are often stockpiled, threatening the environment with spontaneous combustion [116]. Powdered sugarcane bagasse has been found to have a mean particle size of 105.30 μm, a surface area of 4.105 m^2^/g, pore diameter of 2.23 nm, and pore volume of 0.005 cc/g [117]. Due to the significant content of cellulose in bagasse, it is used in various paper types, construction boards, panels, insulating boards, and particleboard production and due to a high content of pentosans it is utilized to obtain chemicals, e.g., lactic acid. Moreover, by fermenting the bagasse or adding manure the biogas of approximate caloric value of 5500 kcal/m^3^ can be produced, which may be used to power petrol or diesel engines. Bagasse is also used to manufacture biodegradable plastic (PHB), agriculture mulch, mushroom subsoil, and ethanol via simultaneous saccharification-cum-fermentation by enzymatic or acid hydrolysis [110]. Bagasse may be hydrolyzed to obtain 85–95% xylose and small percentages of arabinose and glucose. In China and Brazil xylitol is made of bagasse using the reduction process [118]. Due to bagasse’s significant polysaccharide content, it may be processed into insoluble, neutral in taste and odor dietary fiber if processed using alkaline hydrogen peroxide. This process is accompanied by stirring which reduces the content of lignin by about 50% and increases water holding capacity by about 50%, the reason being mechanical shear, which opens fiber structure and makes cellulose hydroxyl groups bind with water [112]. Another method of dietary fiber production is by using sodium hydroxide (NaOH), shown in Figure 3 [119]. Furthermore, sugarcane dietary fiber has been found to be efficient as a gelling agent, generating gels of high strength and displaying a remarkable capacity to hold water [120]. This is especially true when the fiber is present in high concentrations (6 percent). According to Zhuang et al. [121], sugarcane insoluble dietary fiber increased the quality of myofibrillar protein gels by strengthening its structure, increasing the stability of the gel network and reducing its syneresis. Sugarcane dietary fiber may be used to fortify bread [122].

Another byproduct of the sugarcane industry is black strap molasses—a thick liquid of significant viscosity. Molasses is rich in sugar, which further crystallization is not profitable and is not usually meant for direct human consumption due to its chemical composition and unappealing, dark color. About 23–28 L of molasses is generated for every ton of crushed sugarcane. Molasses consists of 45–55% fermentable sugars—30–35% sucrose, 10–25% glucose and fructose, 2–3% non-sugar compounds, water, and minerals. Molasses is used as an ethyl alcohol production substrate during yeast fermentation, then ethyl alcohol is utilized in other chemicals’ production, e.g., ethyl benzene, ethylene oxide, propionic acid, mono chloroacetic acid, their salts, Acetic acid, Beta picoline (3-Methyl Pyridine), Styrene, or Dibutyl phthalate. Ethanol is also used as a fuel oxygenator with 5% concentration—5–20% as a blend with gasoline (Gasohol), or F-95% as fuel extender/replacement. During yeast fermentation for alcohol, carbon dioxide is produced as well, which can be utilized as a cooling agent or to carbonate beverages. As a component of the subsoil for *Aspergillus niger*, a producer of citric acid, baker’s and food yeast, monosodium glutamate, and substitute for coffee, molasses is implicitly utilized in the food industry. Molasses can be used as a compound of fodder, which benefits the microflora of ruminants’ stomachs and helps them digest fibrous feed such as straw. Moreover, fodder enriched with molasses inhibits the development of bronchial disease. It is also used to obtain itaconic acid—a plasticizer and a chemical intermediate; acetone and butanol; dextran—a blood plasma expander and a toothpaste, paint, glue, iron-dextran complex (a medicine for anemia), sulfate dextran (anticoagulant) ingredient; ephedrine—a cough syrup ingredient; biocides; Nitromiel—an explosive; potassium salts; denaturants; activated carbon; asphalt; cement; drawing lubricants; dehydrating agent in mineral clarifying processes; sealing agents [109].

Press mud cake (or press mud) is a leftover residue produced during the filtration of sugarcane juice. It consists of 50–70% moisture, 5–14% crude was and fat, 15–30% fiber, 5–15% sugar, 5–15% crude protein, and a notable deal of Si, Ca, P_2_O_5_, MgO, Fe, and Mn. Press mud is rich in phosphorus, and thus is used as a fertilizer, increasing yield of sugarcane. The mixture of molasses and press mud is utilized as a fertilizer and is useful in animal feed production. Press mud is also used to produce n-triacontanol—a plant growth regulator; policosanol—higher aliphatic alcohol; cement; distemper paints; foaming agents; activated carbon; filter aids; proteins; carnauba wax replacement [109].

During the processing of sugarcane, a significant amount of foliage is generated. Furthermore, almost all of the leftover sugarcane green tips are converted into cattle feed, which is an inexpensive and wholesome source of feed. Dried leaves are used as a fertilizer or may be ground and utilized as a filler in plastics and linoleum production [109].

### 2.5. Acorn

**Figure 4:**
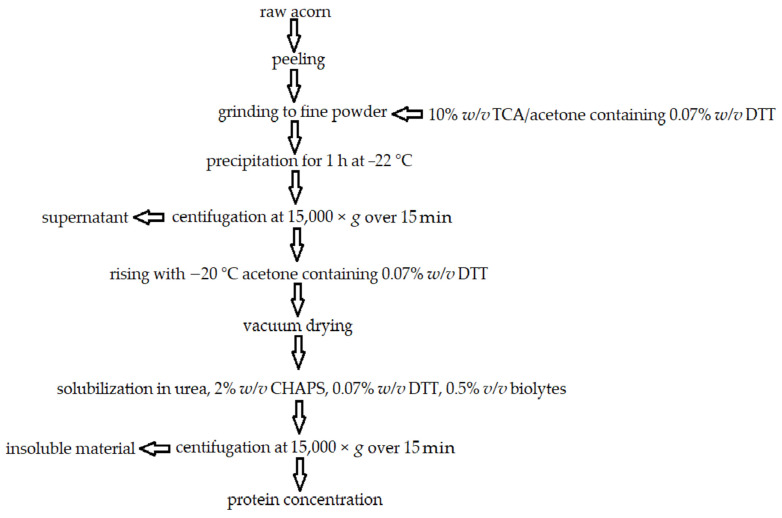
Flow chart of acorn protein extraction. Own figure based on Ref. [133]. 2022, Galván et al.

“Acorn” is a common name for the fruit of plants of the genus *Quercus* (oak trees), belonging to the family Fagaceae. The genus *Quercus* grows in the USA, temperate Europe, Asia, and subtropical Africa [123,124,125]. Acorns have been present in the human diet for ages being used, e.g., as flour in bread craft [126]. Nowadays, acorns are sometimes used in Mediterranean countries in times of food scarcity or as an ingredient of traditional beverages such as Raccahout (Turkish drink resembling hot chocolate), Eichel Kaffee (acorn coffee), or Licor de Bolota (Portuguese alcoholic drink) [127]. These fruits grow in the wild, most often being unused and their valuable functional ingredients such as proteins, carbohydrates, and lipids are wasted. By gathering and processing, such compounds can be of use in the food industry [128]. Acorns may also be used as hog feed because of their notable content of macronutrients. Oils isolated from acorns exhibit resemble olive oil in terms of color, iodine value, UV extinction, coefficient, fatty acid composition, and refractive index [129,130]. Acorns contain a significant amount of unsaturated fatty acids (60% of oleic acid, ω9, and 16% of linoleic acid, ω6), fiber, chlorophylls, carotenoids, phenolic compounds [127], typically 2–5% proteins, vitamins A and E, minerals—P, K, Ca, and Mg, high amount of glycine, lysine, and proline thus, being more nutritious than many cereals. Acorns may be exploited as a source of new hydrocolloids in the food sector due to their high starch content (approximately 50% greater than cereals) and fiber content. Moreover, acorn starch exhibits high paste consistency thus, may be utilized to thicken food and as a stabilizing agent. Acorn protein emulsifying properties come from lysine, which linear structure is believed to act like a potent surfactant at its isoelectric point [128]. Utilization of plant proteins, including acorn protein, as emulsifiers, stabilizers, or foaming agents is a novel approach to food texture modifications [131,132]. Acorn protein has been proven effective as an emulsifier in oil/water environments with a protein concentration of 0.5–2% (*w*/*v*). Acorn protein reduces the viscosity of o/w emulsions [128] Suggested acorn taxa for protein extraction is *Q. infectoria* spp. *boissieri*, because it contains the most protein (8.44%) among other acorn taxa. Acorn protein extraction was presented in Figure 4.

### 2.6. A New Approach to Gelatin

Gelatin is made by breaking cross-linkages between the polypeptide chains and bonds in the parent protein collagen, obtaining a heterogeneous mixture. Even further treatment of enzymes yields gelatin hydrolysates, which show pro-health benefits [134,135,136] and can be used as functional ingredients to provide cryoprotective effects helpful with food exposed to freeze-thaw cycles [137]. According to FAOSTAT [138], poultry meat production increased by about 35% between 2010 and 2020, resulting in corresponding byproduct production. The yearly manufacture of gelatin is about 375,000–400,000 tons [139], of which only 2% is not from mammals [140]. 

Despite its wide range of applications, gelatin concerns some consumers because of religious (haram or not kosher food) and health (possibility of prion disease in bovine gelatin) reasons. To overcome those issues and make use of meat and fish byproducts, it is suggested to produce gelatin from poultry skins, feet, heads, and bones and process waste of the fishery industry [141,142,143]. The gelatin yield depends on the amount of collagen present in a byproduct, the least abundant being the poultry head and feet (28% wet basis) and the most abundant fish skins, bones, and fins (33% wet basis) [144]. The most common fish used for gelatin production are Atlantic salmon, cod, sin croaker, short fin scad, Alaska pollock, big eye snapper, brown stripe red snapper, yellow-fin tuna, Nile perch, black and red tilapia, grass, and silver carp [145]. When gelatins from several aquatic animal species are merged, new properties emerge, allowing for a wider range of applications in the food industry [146]. Compared to traditional bovine gelatin, gelatin from cold-water fish, e.g., cod, megrim, tuna, and tilapia exhibits lower gelling and melting temperatures and similar gel strengths because of lower hydroxyproline and proline, the content of the amino acids. Amino acid content decreases as the environment of the fish is colder. Cold-water fish gelatin requires chemical or enzymatic modifications to be effective in commercial use, or its utilization would be limited to refrigerated products. The gelatin obtained from warm fish byproducts exhibits similar physicochemical properties to porcine or bovine gelatin, and thus, may replace them without significant modifications [147]. Another drawback of fish gelatin is its unpleasant, fishy odor [148]; however, it can be almost entirely neutralized by sulfuric acid, citric acid, and sodium hydroxide treatment. Moreover, such a procedure dramatically increases the gel’s clarity [149]. Porcine skin gelatin exhibits higher foam capacity and foam stability compared to shark cartilage and precooked tuna fin gelatin [150,151]. In the case of protein films, gelatin from channel catfish and Nile perch exhibit film strength, tensile strength, percentage of strain, and water vapor permeability comparable with mammal-derived gelatin [152,153]. A comparison between various aquatic animals-derived gelatin has been provided in Table 4. Although gelatin is not a novel hydrocolloid, its production from poultry and fishery byproducts is innovative.

**Figure 5:**
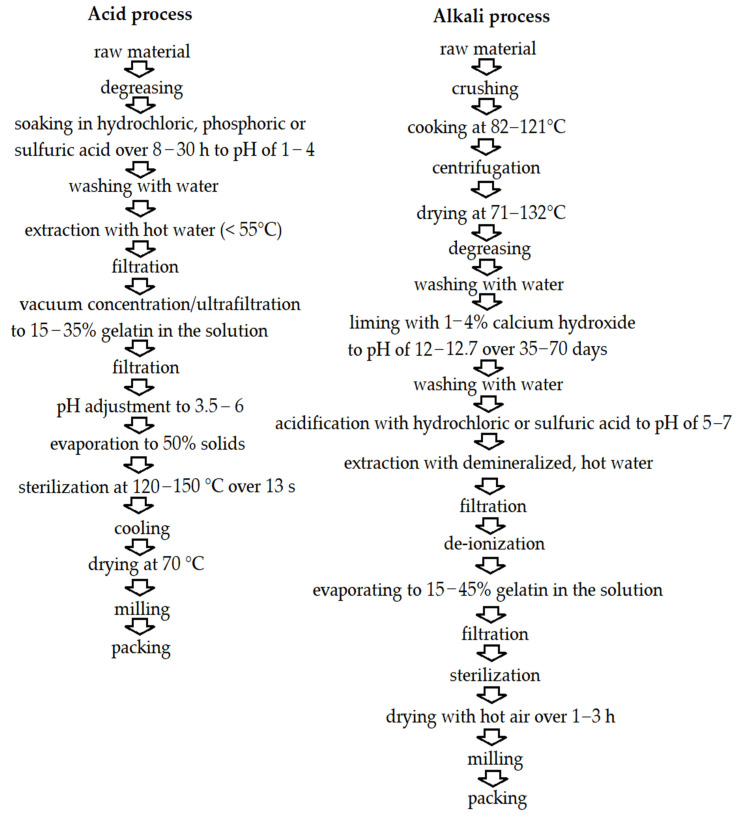
Flow diagram of acid and alkali process of gelatin production. Own figure based on Ref. [146]. 2022, Wasswa et al.

The process of acid and alkali gelatin extraction from fishery byproducts has been shown in Figure 5.

## 3. Health-Promoting Properties of Waste Gelatin from the Fish and Poultry Industry and Byproducts from Arrowroot, Kuzu, Sassafras Tree, Sugarcane, and Acorn

It is important to note the health advantages of arrowroot starch. It is characterized by high digestibility and high content of dietary fibers; contains raffinose, lactulose, stachyose, and fructooligosaccharides which might serve the purpose of prebiotics [19], meaning they promote the growth of favorable bacteria such as *Bifidobacterium* and *Lactobacillus* in the large intestine without stimulating the harmful bacteria such as *Clostridium perfringens.* As a result, humans absorb microelements including Ca, Mg, and Fe more readily and are less likely to develop diseases such as large intestine cancer and disorders brought on by excessive cholesterol levels. Presently, in the large intestine, the prebiotics ferment, producing short-chain fatty acids, which can limit the development of pathogens [168]. As a result of the starch’s anti-inflammatory and anti-irritating effects, it is used to alleviate tissue and bowel disease [169]. This seems especially meaningful regarding ecology and human safety since traditional drugs cause a variety of side effects and may interfere with the environment. Arrowroot starch nanocrystals do not cause such harmful effects [170]. The rhizome of arrowroot is safe for those with phenylketonuria since it is high in alkaloids, glucosides, phenolic compounds, terpenoids, saponins, flavones, tannins, [171] phosphorus, sodium, potassium, magnesium, iron, calcium, and zinc, and has a medium level of phenylalanine. A rhizome is also known for immunostimulatory—significantly increased IgG, IgM, and IgA levels in mouse serum [31]. Arrowroot starch shows antioxidant properties [21] most likely by trapping peroxyl and hydroxyl radicals [172]. This leads to the mitigation of diabetes, cardiovascular disease, high blood pressure, and cancer control [173]. Its short fibers are easy to digest, making it useful for baby diets and children with autism or down syndrome [174]. Arrowroot is also a source of type III resistant starch, proven beneficial for health as a dietary fiber fraction and a valuable food processing element [175]. Another benefit of arrowroot starch is a very low glycemic index of 14 [24]. Foods with a low glycemic index (GI) are more favorable compared to those with a high GI, considering health issues. A diet composed of low-GI foods help maintain proper body weight, body fat, manage hyperlipidemia, and diabetes [176]. Although arrowroot is mainly used for starch production, many works point out its medical applications [22]. Arrowroot is abundant in a good deal of healthy substances, e.g., alkaloids, steroids, phenolic compounds, and flavonoids. Solvents and found compounds are presented in Table 5. Moreover, arrowroot leaves may extract antidiarrheal compounds [177].

Kuzu is known for its use in diabetes effects mitigation due to the significant content of isoflavonoids, especially puerarin, known for its ability to restore glucose balance. [116]. Pueranin is used against migraines as well because it regulates cerebral blood circulation [180]. Kuzu is also used in the treatment of flu, fever, nausea, allergies, and diseases of the upper respiratory tract. Moreover, kuzu has a significant alkaline effect that is useful in deacidifying, detoxifying, and regulating the body’s metabolism. Intake of kuzu increases the content of happiness hormones–serotonin and dopamine, which are responsible for maintaining a positive mental state and preventing stress. It also lowers blood pressure, increasing the economization of heartbeat and reducing the risk of a heart attack. The consumption of kuzu may also help stimulate the immune system by penetrating the human intestines to cope with bacterial infections and suppress smooth muscle contractions [77]. Furthermore, kuzu root contains bioactive isoflavones: isoflavonoid glucosides, coumarins, puerarols, but-2-enolides and their derivatives [181], daidzein, daidzin, puerarin, formononetin-7-O-glucoside (ononin), 3-methoxypuerarin, 6-O-D-xylosylpuerarin, 3-methoxydaidzein, genistein, biochanin A, formononetin and isoflavone glucosides, e.g., daidzein 8-C-apiosyl-(1→6) glucoside. These compounds exhibit hepatoprotective [182], antioxidant [183], anti-diabetes [184], neuroprotective [185], cardiovascular protective [186], anti-inflammatory [187], estrogenic [188], antineoplastic, antiatherogenic, antiarrhythmic, antihypertensive, detoxifying, and diuretic activities. However, the mentioned substances exhibited different antioxidant properties than expected, suggesting that more research is needed [189]. Puerarin can dilate blood vessels, which decreases blood pressure [180]. Daidzein, also known as phytoestrogen, helps with alcoholism prevention, reducing the urge to alcohol consumption by up to 80%. Daidzein is also known for its antioxidant properties, alleviating the consequences of alcohol intoxication, and helping heal organs already damaged by alcohol. Intake of kuzu also helps with coping with nicotine addiction. The great advantage of kuzu therapy is the lack of side effects [81]; however, Wong et al. [180] state that there are no regulations regarding contaminants in kuzu root, such as excessive or banned pesticides, microbial contaminants, heavy metals, and chemical toxins.

Sassafras leaves contain many essential oils, including geranial, neral, limonene, caryophyllene, α-pinene, (Z)-3-hexenol, linalool, the caryophyllene oxide [190]. In traditional medicine, sassafras infusions are used to treat colds, high blood pressure, heart troubles, swelling, worms, fever [191], stomach ache, urinary retention, scurvy, jaundice, pregnancy difficulties, cancer, typhus, dropsy [192], diarrhea, rheumatism, measles, scarlet fever, burns, lower chest pain, nausea, vomiting, indigestion, constipation, loss of appetite, gallstones, bladder pain, or as a blood purifier [193]. Formerly, boiled sassafras leaves were used as an abortifacient [194].

Sugarcane bagasse and sugarcane tops show promising pro-health benefits if processed, to dietary fiber using alkaline (NaOH or H_2_O_2_) treatment. Dietary fibers from both bagasse and sugarcane used in the food industry demonstrated significant nutritional value. However, H_2_O_2_ treatment promoted oxidation and free radical occurrence thus, being threatening for some human food macromolecules. In terms of chapatti-style bread and pasta noodles, an inclusion of no more than 8% of these fibers has been deemed agreeable [119]. The main pro-health characteristics of dietary fibers are inhibition of carbohydrate and fat digestion, which helps deal with diabetes [195]; hyperglycemia control [196]; diabesity prevention; inflammation control; Alzheimer’s disease and vascular dementia prevention; depression and anxiety mitigation; hypocholesterolemic effect; lowering the blood pressure; cardiovascular disease prevention; colon cancer prevention [197]. Dietary fiber is known to have even more health-promoting properties; however, dietary fiber benefits are not the main topic of this work.

Acorn protein may provide some health-promoting benefits. Acorn proteins consist, among others, of legumin, legumin precursors, which show antioxidant activities, and arterial pressure regulating properties, by inhibiting I-converting enzyme [198]; 2-Cys peroxiredoxin and peroxiredoxin-2b proteins, both responsible for mitigating the oxidative stress in plants thus, most likely serving an antioxidative purpose; chitinase being a protein responsible for defensive mechanism against pathogens [140] thereby, inhibits the growth of fungi and helps with cancer prevention [199]. Acorn protein has a significant amount of leucine, isoleucine, and threonine presented in Table 6. However, acorn protein alone cannot provide a sufficient number of amino acids [200]. Mentioned compounds are not synthesized in the human body and need to be supplied with food for proper health. Lack of indispensable amino acids in the human diet results in several health conditions such as depression, anxiety, insomnia, fatigue, weakness, and growth stunting in the young. The more severe consequences of indispensable amino acids deficiency are kwashiorkor–a state of malnutrition manifesting as peripheral edema, dry peeling skin with hyperkeratosis and hyperpigmentation, ascites, liver malfunction, immune deficits, anemia, and relatively unchanged muscle protein composition; and marasmus–severe physical wasting [201].

It is possible to obtain fish and aquatic animal gelatin hydrolysates using various proteolytic enzymes. Gelatin hydrolysates and gelatin-derived peptides show health-promoting properties [15], shown in Table 7.

## 4. Conclusions

Many food industries focus on manufacturing products, which are allegedly the most profitable and easiest to produce, discarding the byproducts into landfills or directly to the environment polluting it. This causes additional transportation and storage costs. Moreover, pollution poses a threat to wild animals and humans as well. Fortunately, production waste has a lot to offer to manufacturers, since it is most often loaded with useful materials, functional substances, and health-promoting compounds. Byproducts such as poultry feet, beaks, feathers, skin or fish scales, fins, heads, and bones are typically discarded or processed into fodder. However, such a common ingredient might be used in a novel way to produce safe gelatin and a substrate for gelatin hydrolysates that are beneficial for health. Many plants are cultivated for their medical application. After processing, leftovers are discarded. These byproducts frequently contain valuable, functional compounds such as foaming agents, surfactants, gelling and thickening agents, cryoprotectants, or syneresis inhibitors. Moreover, the byproducts often pose medical applications too. They may exhibit antioxidative, nutritional, anti-cancer, diabetes regulating, or prebiotic activity. It is worth considering the economic strategy used by sugarcane manufacturers, who not only produce sugar but also utilize the waste to generate fuel for their units, fodder for locals, and fertilizer for their plants. Such technology may be introduced to more food processing industries such as starch, meat, grain, or plant-derived bio-compounds industries. Doing so can reduce the cost of production, transit, and storage, therefore, providing consumers with lower prices without losses for the manufacturers.

## Figures and Tables

**Table 1:** Arrowroot rhizome starch composition. N/E—not evaluated.

Compound	Moisture (%)	Fat (%)	Protein (%)	Carbohydrates (%)	Ash (%)	Soluble Fiber (%)	Insoluble Fiber (%)	Reference
Starch	11.90	0.84	0.14	rest	0.58	5.00	8.70	[31]
	15.24	0.01	0.40	83.91	0.33	N/E	N/E	[30]
	10.2	N/E	0.6	84.2	N/E	N/E	N/E	[32]
	7.06	1.43	3.75	80.77	3.60	3.96		[33]

**Table 2:** Amylose and amylopectin composition of arrowroot rhizome starch. N/E—not evaluated.

Polysaccharide	Amylose (%)	Amylopectin (%)	Reference
Starch	21.9	62.3	[32]
	22	N/E	[34]
	19.0–19.9	N/E	[35]
	15.21	84.79	[36]
	24.8	N/E	[37]
	>40	N/E	[38]
	20	80	[18]

**Table 3:** Composition of kuzu starch isolated from its roots.

Ingredient	Starch as Dry Basis (%) *w/w*	Amylose (%)	Amylopectin (%)	Reference
root	51.6	N/E	N/E	[65]
	15–35	19–24	20.5	[50]
	N/E	22.2–23.34	N/E	[66]

**Table 4:** Aquatic animals derived gelatin compared.

Source	Yield (Wet Basis)	Bloom/gel Strength	Reference
Atlantic salmon skin	4–11.3%	80–108 g	[154]
Atlantic cod skin	44.8% ^c^	71 g	[154]
Bigeye snapper skin	6.5%	105.7 g	[155]
Bigeye snapper skin	40.3% ^a^	138.6 g	[156]
Brownbanded bamboo shark	19.06–22.81%	56.53–217.26 g	[157]
Blacktip shark	21.17–24.76%	10.43–207.83 g	[157]
Black tilapia skin	5.39%	181 g	[158]
Bigeye snapper skin	6.5%	105.7 g	[155]
Channel catfish	19.2% ^b^	252 g	[159]
Cod skin	17%	180 g	[149]
Cuttlefish skin	36.82% ^c^ (dorsal skin) and 59.69% ^c^ (ventral skin)	126 g (dorsal skin) and 137 g (ventral skin)	[160]
Giant catfish skin	20.1%	153 g	[161]
Giant squid inner and outer tunics	12%	147 g	[162]
Grass carp	11.3% ^a^	N/E	[163]
Lumpfish skin	14.3%	N/E	[164]
Megrim skin	10%	360 g	[165]
Nile perch bone	2.4%	134–160 g	[152]
Nile perch skin	16%	134–229 g	[152]
Pollock skin	18% ^b^	460 g	[136]
Red tilapia skin	7.81%	128 g	[158]
Shark cartilage	17.34%	111.9 kPa	[150]
Shortfin scad skin	7.25%	177 g	[14]
Sin croaker skin	14.3%	125 g	[14]
Silver carp skin	11% ^a^	600 g	[166]
Tilapia skin	N/E	263 g	[149]
Tuna fin	1.25%	126 g	[151]
Yellowfin tuna skin	89.7%	426 kPa	[167]

^a^—based on the hydroxyproline content of the gelatin in comparison with that in the skin. ^b^—based on the protein content of the gelatin in comparison with the wet weight of raw material. ^c^—dry weight. N/E—not evaluated.

**Table 5:** Solvents and soluble compounds of arrowroot extracts.

Compound	Reference					
	[178]		[179]			
	Methanol	Water	Ether	Chloroform	Methanol	Water
alkaloids	P	P	N	N	P	N
steroids	P	N	P	P	N	N
phenolic compounds	P	P	N	N	P	P
flavones	N/E	N/E	N	N	P	N
flavonoids	P	N	N	N	N	N
flavonones	N/E	N/E	N	N	P	N
glycosides	P	P	P	P	P	P
saponins	P	P	P	P	P	N
terpenoids	P	P	P	P	P	N
tannins	P	N	N	N	P	N

P—present, N—not present, N/E—not evaluated.

**Table 6:** Aminogram of acorn *(Quercus rotundifolia*) kernel protein. According to [202].

Amino Acid (AA)	Protein Content (g AA/ kg Protein)
**Essential amino acids**	
arginine	65
lysine	43
histidine	18
isoleucine	47
leucine	62
methionine	22
Methionine + cystine	45
phenylalanine	45
Phenylalanine + tyrosine	64
threonine	32
valine	58
**Non-essential amino acids**	
Aspartic acid	205
Glutamic acid	143
serine	42
glycine	43
alanine	46
proline	65
tyrosine	26
cystine	23

**Table 7:** Health benefits of aquatic animal gelatin hydrolysates and gelatin-derived peptides with used enzymes.

Fish or Aquatic Animals	Enzyme Used	Pro-Health Benefits	Reference
Alaska pollock skin	Pronase E	Antioxidant	[203]
Atlantic salmon skin	Flavourzyme	Dipeptidyl-peptidase IV enzyme inhibitory activity–type 2 diabetes, symptoms mitigation	[204]
Amur sturgeon skin	Alcalase	Antioxidant, cryoprotective benefit	[205]
Brownstripe red snapper skin	Trypsin-like proteases from pyloric caeca	Antioxidant	[206]
Blacktip shark skin	Papain, papaya latex crude enzymes	Antioxidants, hypertension prevention, human LDL cholesterol inhibition, DNA oxidation inhibition, metal ion chelation	[207,208,209,210]
Chum salmon skin	Papain, Alcalase	Cell proliferation, cycle progression, apoptosis	[211]
Hoki skin gelatin	Trypsin	Antioxidant	[212]
Japanese flounder skin	Pepsin	Antioxidant	[213]
Jumbo squid skin	Trypsin	Antioxidant	[214]
Nile tilapia scale	Alcalase	Antioxidant	[215]
Pacific cod scale	Pepsin, trypsin, α–chymotrypsin	Antioxidant, antihypertensive benefit	[216]
Pacific cod skin	Papain	Antioxidant, ACE-inhibition (hypertension prevention)	[208]
Squid inner and outer tunics	Protamex, trypsin, neutrase, savinase, NS37005, esperase, alcalase	Antioxidant, hypertension prevention, anticancer benefit against lines MCF-7 and U87	[217]
Squid skin	Pepsin	Hypertension prevention	[218]
Tilapia skin	Properase E, multifactor neutral	Antioxidant, photoaging prevention	[219,220]
